# Association of active coping to unfair treatment with perceived stress and depressive symptoms in African Americans: mh-grid study

**DOI:** 10.1186/s12888-022-03772-y

**Published:** 2022-02-21

**Authors:** Ayomide R. Ojebuoboh, Amparo G. Gonzalez-Feliciano, Kristen M. Brown, Rumana J. Khan, Ruihua Xu, Lisa A. DeRoo, Jessica Lewis, Rakale C. Quarells, Sharon K. Davis

**Affiliations:** 1 University of Minnesota Medical Scientist Training Program, 420 Delaware St SE, Minneapolis, MN 55455 USA; 2 Social Epidemiology Research Unit, National Institutes of Health, National Human Genome Research Institute, Bethesda, USA; 3 Cardiovascular Research Institute, Morehouse School of Medicine, Atlanta, GA USA

**Keywords:** Unfair treatment, Depressive symptoms, African American health, Perceived stress, Talking, Active coping

## Abstract

**Background:**

Unfair treatment such as discrimination and racism contribute to depression and perceived stress in African Americans. Although studies have examined how responding to such treatment is associated with ameliorating depressive symptoms and levels of perceived stress, most do not focus on African Americans. The purpose of this study is to assess how talking to others in response to unfair treatment is associated with self-reported depressive symptoms and perceived stress levels in African Americans.

**Methods:**

A sample from the 2010–2013 Minority Health Genomics and Translational Research Bio-Repository Database was used and consisted of 376 African American adults aged 30–55 years old residing in the southern region of the United States. Linear regression models were used to assess the association between talking to others following unfair treatment, compared to keeping it to oneself, on self-reported depressive symptoms and perceived stress. The predictor variable was based on the question “If you have been treated unfairly, do you usually talk to people about it or keep it to yourself?”.

**Results:**

Talking to someone after being treated unfairly was inversely associated with perceived stress ($\beta$: -3.62, SE: 1.14, p ≤ 0.05) and depressive symptoms ($\beta$: -3.62, SE: 1.14, p ≤ 0.05).

**Conclusions:**

African Americans who talked to others in response to unfair treatment had lower depressive symptoms and perceived stress than those who kept it to themselves. More outreach to African Americans regarding the importance of talk in response to exposure to unfair treatment is needed as a potential coping mechanism.

**Supplementary Information:**

The online version contains supplementary material available at 10.1186/s12888-022-03772-y.

## Background

African Americans continue to face disproportionate experiences with unfair treatment, racial discrimination and unequal access to healthcare and other necessary resources [1–5]. This negative impact on the health of African Americans includes overall lower quality of life and high prevalence of low-quality sleep and narcolepsy, which is associated with biological dysregulation and subsequent allostatic load [6, 7]. African Americans exposed to mistreatment due to perceived racial discrimination have a higher average diastolic blood pressure reactivity compared to those who did not face mistreatment due to perceived racial discrimination [8]. Since stress due to discrimination plays a role in the physical and mental health of African Americans, it is important to examine methods that can alleviate perceived stress in African Americans [9].

In addition to increased perceived stress, African Americans also have a higher likelihood of suffering with more severe and long-term depression [10, 11]. There are many factors including discrimination, socioeconomic status, systemic racism in the medical community and stressful life events that contribute to depression in African Americans [11].Since depressive symptoms can be a precursor for the future development of Major Depressive Disorder (MDD), it is important to determine ways to ameliorate depressive symptoms in African Americans order to prevent the chronicity of MDD.

Research has explored different coping mechanisms used to reduce perceived stress in African Americans like interpersonal support, disengagement coping and emotion-focused coping [12–15]. There are many studies that have examined how African Americans cope with racism and discrimination [16–19]. Although racism and discrimination are a form of unfair treatment, other papers have focused more on coping mechanisms in response to racism and discrimination and not unfair treatment per se. The John Henryism scale, which represents high-energy/high effort coping, is an effective tool that has been used to show how certain coping methods can alleviate stress among African Americans [16]. This scale examines three themes: 1) efficacious mental and physical vigor, 2) a strong commitment to hard work and 3) a single-minded determination to succeed [16]. Previous research has shown the association between John Henryism and systolic blood pressure reduction for African American men [20]. It can also be a moderator for the association between race-related stress and rumination [16]. Additionally, disengagement coping has been shown to partially mediate the association between depressive symptoms and the Strong Black Woman Complex Africultural Coping Systems Inventory is another measure that has been used to represent culture-specific coping strategies that African Americans use during stressful situations [21]. Prior research has also shown that spiritual well-being can partially mediate the association between Africultural Coping Systems Inventory, and quality of life [22].

While there is more focus on examining coping mechanisms for stress, there is more emphasis on examining treatment options for depression, like antidepressants, electroconvulsive therapy (ECT), selective serotonin reuptake inhibitors (SSRIs), and psychotherapy [23]. Psychotherapy, which is also called talk therapy, has shown strong evidence of reducing depressive symptoms [24]. However, many of the discussions on psychotherapy have focused on African Americans’ perspective on psychotherapy, instead of how it could be used as a strategy in alleviating depressive symptoms [25]. Encouraging social support is another way to address both depressive symptoms and perceived stress levels in African Americans, as evidence shows social support plays a positive role in lessening perceived stress and depressive symptoms in African Americans [26, 27]. Specific types of social support like emotional and instrumental support can also lead to different outcomes on perceived stress [28]. While emotional support consists of going to friends and family about one’s problems, instrumental support involves family and friends completing actions [28].

Although social support has been studied extensively among African Americans, most studies have examined overall social support and not the association of talking which is emotional support [29, 30]. The focus of this project differs in that we focus on examining the difference between talking to others about unfair treatment compared to keeping it to oneself as the predictor for coping. The purpose of this study is to examine the association between 1) talking to someone in response to unfair treatment and perceived stress and 2) talking to someone in response to unfair treatment and depressive symptoms in a cohort of African Americans. We will also examine the association between perceived stress and depressive symptoms. We hypothesize that there would be a negative association between talking and depressive symptoms, in addition to a negative association between talking and perceived stress levels. Investigating the influence of talking after unfair treatment as a coping mechanism can yield important insight as a potential mechanism to ameliorate chronic disease risk factors associated with stress and mental health conditions that disproportionately affects African Americans. Although talking should not replace treatments for depression, it could be an additional method incorporated in coping mechanisms to alleviate perceived stress and treatments for depressive symptoms in African Americans.

## Methods

Data for this study were obtained from The Minority Health Genomics and Translational Research Bio-Repository Database (MH-GRID) study which was a multi-cohort case–control study of African Americans aged 30–55 years with optimal blood pressure and severe or resistant hypertension from the southern region of the United States. Details of the MH-GRID study design are described in detail elsewhere [31]. In this study, we focused on a subset of MH-GRID participants who were recruited from Morehouse School of Medicine (Atlanta, GA), Kaiser Permanente-Georgia (Atlanta, GA) the Grady Health System (Atlanta, GA) and the Jackson Hinds Clinic (Jackson, MS) between April 2012 and September 2013. Participants in MH-GRID signed written informed consent prior to participation in the study [31]. The original sample consisted of 490 participants, but after excluding participants with missing data, the sample for this study included 237 women and 139 men for a total of 376 participants. This study received approval from the Morehouse School of Medicine, Kaiser Permanente and the National Institutes of Health Institutional Review Boards, in addition to the Grady Health System Research Oversight Committee.

### Predictor variable

The unfair treatment variable was derived from Krieger et. al’s Experiences of Discrimination Questionnaire [32]. Response to unfair treatment was the primary predictor variable based on the yes/no question “If you have been treated unfairly, do you usually talk to people about it or keep it to yourself? The reference was “keep it to yourself”. The response ‘Talk to people about it’ is considered active coping, and “keep it to yourself” is considered passive coping based on a similar study that used a similar classification [17]. The ‘don’t know/not sure’ and ‘refused’ response categories were excluded from the analyses. Response to unfair treatment was also used as the primary predictor variable based on the question “If you have been treated unfairly, do you usually accept it as a fact of life about it or try to do something about it?” However, the results were insignificant, so they were not displayed in the analyses.

### Outcome variables

The 20-item Center for Epidemiologic Studies Depression Scale (CES-D) was used to assess the presence of depressive symptoms [33]. CES-D Scale is a self-report instrument used to measure several functional domains commonly linked to depression [33]. The scale has been found as a reliable measure for assessing depressive symptoms in large-scale epidemiological studies. Participants indicated how often over the past week, they experienced each of the 20 symptoms described in the CES-D scale. Responses were made on a 4-point scale ranging from 0 (rarely or none of the time) to 3 (most of or all the time)**.** After reverse coding for 4 items, higher scores indicate greater frequency of depressive symptoms. The Cronbach Alpha’s score for the CES-D is 0.83.

The Cohen’s 14-item Perceived Stress Scale (PSS) was used as a measure of perceived stress which consisted of 14 questions related to the participant’s level of exposure to perceived stress in the last month [34]. The PSS was administered by trained health professionals. Participants had to choose from 1 = never, 2 = almost never, 3 = sometimes, 4 = fairly often and 5 = very often. Each item used the Likert scale from 1–5, but were relabeled to 0–4 for analytical purposes. The scale was calculated by first reversing the numbers for relevant positive variables [35]. Then, all the 14-items were summed for each participant**.** A high PSS value equated to higher stress and a low PSS value equated to lower stress. The Cronbach Alpha’s alpha score for the PSS is 0.91.

### Covariates

Covariates included age, sex, level of education, marital status, employment status and hypertension status. Covariates used for the adjusted models were chosen based on their possible relationship with the outcome variables. Sociodemographic variables were based on self-report at the time of enrollment and hypertension was defined as a systolic blood pressure of between ≥ 140 mm Hg and ≤ 180 mm Hg, diastolic blood pressure between ≥ 90 mm Hg and ≤ 110 mm Hg, or the use of ≥ 2 medications for blood pressure for at least the last 3 months. Self-reported sex and age was recorded at baseline. Education was divided into three categories: 1) ≤ 12 years or graduate equivalency diploma (GED), 2) some college/technical school and 3) college graduate or higher. Those who were classified as married included those who were separated, living with a partner and married. Those who were unmarried included participants who were widowed, divorced, or single/never married. The employed group consisted of participants who were working part-time, on leave for sick reasons, and working full time. Unemployed included participants who were retired, homemakers, looking for work, students and employed, but temporarily laid off.

### Statistical analysis

Linear regression models were used to calculate the unadjusted and adjusted $\beta$ estimates for the association of talking following unfair treatment compared to keeping it to oneself on perceived stress and depressive symptoms. Linear regression models were also used to calculate the unadjusted and adjusted $\beta$ estimates for the association between perceived stress and depressive symptoms. Sex, level of education, marital status, employment status, and hypertension were entered as categorical variables and age as a continuous variable. Interactions between sociodemographic covariates and talking to other people in response to unfair treatment with both perceived stress and depressive symptoms were tested in adjusted linear regression models separately. Significance was based on a two tailed significance level of ≤ 0.05. A *p* value < 0.10 was also considered as slightly significant because it can also be considered as “trending toward statistical significance” [36]. The analyses were conducted using the SAS software [37].

## Results

Table 1 presents the descriptive characteristics of the study population and shows that the mean age was 45.84 years old. The distribution between those who completed some college/technical college and college graduate or higher was similar, however majority of participants were in the completed ≤ 12 years or GED (36.70%) group. There were also more women (63.03%) than men (36.97%). Most of the participants were unmarried (61.97%). The percentage of those employed was higher than those unemployed (61.70% versus 38.30%, respectively) and 62.50% of respondents had hypertension. Most participants reported talking to someone in response to unfair treatment compared to a lower proportion who kept it to themselves (85.90% vs. 14.10%). The mean depression score was 12.47 and the mean perceived stress score was 21.78. This shows that the mean depression score was along the spectrum of the scale, while the average perceived stress score was among the lower/medium spectrum of the scale.

**Table 1:** Descriptive characteristics of study sample, MH-GRID^a^, *N* = 376

**Characteristics**	**%**	**Mean (std**^**b**^**)**
Age, years		45.84 (6.68)
Sex		
Women	237 (63.03%)	
Men	139 (36.97%)	
Education		
≤ 12 years or GED^c^	138 (36.70%)	
Some college or technical college	114 (30.32%)	
College graduate or higher	124 (32.98%)	
Marital Status		
Unmarried	233 (61.97%)	
Married	143 (38.03%)	
Employment status		
Unemployed	144 (38.30%)	
Employed	232 (61.70%)	
Hypertension, mmHg		
No	141 (37.50%)	
Yes	235 (62.50%)	
How do you respond if treated unfairly?		
Talk to Someone About It	323 (85.90%)	
Keep It To Themselves	53(14.10%)	
Perceived Stress Scale		21.78 (8.70)
Depression Scale		12.47 (10.63)

^a^*MH-GRID* Minority Health Genomics and Translational Research Bio-Repository Database

^b^*STD* standard deviation

^c^*GED* Graduate Equivalency Diploma

Table 2 reveals the unadjusted and adjusted linear regression models for the association between talking following unfair treatment on the perceived stress scale. In the unadjusted model, perceived stress was reduced by 4.08 units among participants who spoke to someone following exposure to unfair treatment ($\beta$: -4.08, SE: 1.14,—*p* ≤ 0.05) and 3.4 units in the adjusted model ($\beta$: -3.62, SE:1.14, p ≤ 0.05)

**Table 2:** Linear regression of the association of talking following unfair treatment on perceived stress in African Americans, MH-GRID^a^, *N* = 376

**Perceived Stress Scale**	**Talking following unfair treatment**^**b**^			
	**Unadjusted**		**Adjusted**^**c**^	
	${\varvec{\beta}}$ **Estimate (SE**^**d**^**)**	***P* value**	${\varvec{\beta}}$ **Estimate (SE**^**d**^**)**	***P* value**
**14-item PSS**^**e**^	-4.08 (1.14)	*p* ≤ 0.05	-3.62(1.14)	*p* ≤ 0.05

^a^*MH-GRID* Minority Health Genomics and Translational Research Bio-Repository Database

^b^Compared to keeping it to themselves

^c^Adjusted model include age, sex, educational level, marital, employment and hypertension status

^d^*SE* Standard Error

^e^*PSS* Perceived Stress Scale

Table 3 reveals that those who talked to someone following unfair treatment, compared to those who kept it to themselves, experienced a lower level of depression symptoms by 5.65 nits in the unadjusted model ($\beta$:-5.65, standard error: 1.55, *p* ≤ 0.05) and 3.75 units in the adjusted model ($\beta$: -3.75, Standard Error: 1.45, *p* ≤ 0.05).

**Table 3:** Linear regression of the association of talking following exposure to unfair treatment on self-reported level of depressive symptoms in African Americans, MH-GRID^a^, *N* = 376

Depression Scale	Talking following unfair treatment^**b**^			
	Unadjusted		Adjusted^**c**^	
	${\varvec{\beta}}$ Estimate (SE)^**d**^	*P* value	${\varvec{\beta}}$ Estimate (SE)^**d**^	*P* value
20-item CES-D Scale^**e**^	-5.65 (1.55)	*p* ≤ 0.05	-3.75 (1.45)	*p* ≤ 0.05

^a^*MH-GRID*  Minority Health Genomics and Translational Research Bio-Repository Database

^b^Compared to keeping it to themselves

^c^Adjusted model include age, sex, educational level, marital, employment and hypertension status

^d^*SE* Standard Error

^e^*CES-D Scale* Center for Epidemiologic Studies Depression Scale

Table 4 shows a positive association between perceived stress and depressive symptoms. In the unadjusted model, as perceived stress increases by 1 unit, depressive symptoms increased by 0.83 units ($\beta$: 0.83, SE: 0.05,—*p* < 0.0001). In the adjusted model, as perceived stress increases by 1 unit, depressive symptoms increased by 0.76 units ($\beta$: 0.76, SE: 0.05,—*p* < 0.0001).

**Table 4:** Linear regression of the association of perceived stress and self-reported level of depressive symptoms in African Americans, MH-GRID^a^, *N* = 376

**Depression Scale**	**PSS**^**b**^			
	**Unadjusted**		**Adjusted**^**c**^	
	${\varvec{\beta}}$ **Estimate** **(SE)**^**d**^	***P* value**	${\varvec{\beta}}$ **Estimate** **(SE)**^**d**^	***P* value**
**20-item CES-D Scale**^**e**^	0.83 (0.05)	*p* < 0.0001	0.76 (0.05)	*p* < 0.0001

^a^*MH-GRID* Minority Health Genomics and Translational Research Bio-Repository Database

^b^Adjusted models include age, sex, educational level, marital, employment and hypertension status

^c^*SE* Standard Error

^d^*PSS* Perceived Stress Scale

^e^*CES-D Scale* Center for Epidemiologic Studies Depression Scale

There was only a slight significant interaction with a *p* value $\le 0.10$ between employment status and the talking variable when the outcome was CES-D symptoms (*p* = 0.0974). Also, there was only a borderline significant interaction with a *p* value ≤ 0.10 between employment status and talking to other people in response to unfair with the outcome for perceived stress scale (*p* = 0.0761). Therefore, descriptive results stratified by employment status and results of linear regression models stratified by employment status are presented in the Additional File 1: Tables S1, S2 and S3.

## Discussion

This study revealed the association between talking with someone in response to unfair treatment on level of perceived stress and depressive symptoms among African Americans. Participants who talked with someone about their unfair treatment had less perceived stress and depressive symptoms. There was also a positive association between depressive symptoms and perceived stress. These findings suggest that talking to someone about experiences of unfair treatment may have a positive association between reducing stress and depressive symptoms in African Americans.

These results align with previous studies that have shown that talking to others about stressors can also help with adjusting to stressors [26]. This was shown to be true among a group of undergraduate students. In this experiment, those that had the option to speak with someone about the stressor had lower levels of perceived stress after being exposed to the stressor compared to those who were not able to speak to anyone [26]. Another study that included talking to someone about worries under the scope of emotional social support concluded that there was an inverse relationship between satisfaction with emotional social support and stress levels among African Americans [38]. Despite these findings, there is mixed evidence on the association between social support and perceived stress levels. In this same study, African Americans who had more emotional social support also had higher stress levels, which may be because people seek out more social support when they are experiencing more stressful circumstance [38]. In a different study, those that sought advice from friends and professionals were in more distress than those who did not ask for advice but had the same problems [28].

Additionally, although the focus of this paper is not on psychotherapy, rather than focusing on the broad scope of social support we focused on one specific aspect of social support–-talking. Many studies have examined the role of social support on depressive symptoms or perceived stress in African Americans, but only a few have solely looked at the role of talking to someone [39–41]. The role of talking on depressive symptoms has been studied before within a sample of pregnant African American women [42]. Those who experienced unfair treatment and kept it to themselves had a greater odd of elevated depressive symptoms, compared to those that talked to others [42]. Those who talked to others while also doing something about it had the lowest odds of elevated depression symptoms [42].

In this project, the results align with those of Ertzel et al., 2012, but differs in that it includes both African American men and women [42]. In regards to perceived stress, a 2009 study showed that social support, which includes reaching out and talking to people, was a mediator for the effects of perceived racism on mental and physical health in African Americans; the study, however, did not assess exposure to unfair treatment [43]. Another study that examined a sample of 4,000 African American and White participants showed that 69% of African Americans would do something and talk to others in response to exposure to stressful racism [44]. In this project, a further step was taken by assessing talking to someone in response to unfair treatment and to examine the association between the variable that represented talking and perceived stress.

In addition, this project showed a significant relationship between perceived stress and depressive symptom among African Americans. Many previous studies have examined this relationship, but there aren’t as many focused on African Americans [45–47]. One study that focused on older African Americans showed a correlation between depression symptoms and perceived stress [30]. However, many studies show an association between certain stressors, like discrimination, on depressive symptoms, but not as many have focused on the association between perceived stress and depressive symptoms among African Americans [48, 49]. The purpose of examining this relationship was to show that creating initiatives to reduce depressive symptoms could also indirectly mitigate perceived stress or creating initiatives to reduce perceived stress could also mitigate depressive symptoms in African Americans. Understanding this relationship helps explain why the results in this study showed an association between talking and both perceived stress and depressive symptoms.

Lastly, this project also revealed a marginal significant interaction between employment status and response to unfair treatment when the outcome was either depression or stress. There is more research on coping and workplace stress than the association between employment status and perceived stress in African Americans [50, 51]. There is also mixed evidence on the association between employment status and depression in African Americans in the literature [52–55]. These differing conclusions on the relationship between employment status and depression don’t explain why the results revealed that African Americans who are employed had lower levels of depression when they spoke to others in response to unfair treatment, but African Americans who were unemployed did not have significantly lower depression levels if they spoke to others in response to unfair treatment. Perhaps, due to other health and social factors, such as lack of access to health care and financial instability, these are bigger contributors to their depression as suggested by a study that showed retired African American men may have greater rates of depression than White men because they could face greater sociocultural barriers [56]. However, this project suggests the need for more research that investigates effective methods to reduce depressive symptoms and perceived stress among unemployed African Americans.

Despite these findings, the study has several limitations including the disproportionate group sizes, the use of other medications, regional effects and the cross-sectional analyses in this study. The sample size of those who keep it to themselves is much smaller than the sample of those who talk following unfair treatment which may result in selection bias (14.7% vs 85.7%). Future research should replicate this study with a larger sample size within the group of people who kept it to themselves after unfair treatment in order to confirm results. There was also no evaluation of whether participants currently attended therapy, engaged in coping mechanisms for stress regularly or were currently using treatments for depression because the variable was not available. The use of antidepressant medication could affect the results. If certain patients were taking medications that may affect their mood, their reduced level of depression could be due to that medication instead of talking to others after unfair treatment. Additionally, there is a lack of generalizability because the findings are based on African Americans residing in the southern region of the United States. Although we expect similar findings among African Americans residing in other regions of the country.

## Conclusions

Overall, the findings suggest that African Americans who reported that they usually talk to others following perceived unfair treatment had lower perceived stress and less depressive symptoms compared with those who reported that they keep the experience of unfair treatment to themselves. With depression levels on the rise in the United States from 2005 to 2015, there is an urgent need for more studies that examine not only depression in African Americans but also create interventions to prevent depression in African Americas [57]. Continually examining ways to reduce the depressive symptoms rates in African Americans could also indirectly reduce MDD disparities that exist in the United States. Talking about unfair treatment with others may also be a mechanism to help mitigate stress-induced adverse health conditions in African Americans. More public resources should be allocated towards outreach to African Americans regarding the importance of talking in response to unfair treatment as a potential coping mechanism. Future research should also examine additional coping mechanisms to unfair treatment and its potential to ameliorate chronic disease risk factors and mental health conditions such as depression and stress in African Americans.

## Supplementary Information

**Additional file 1.**

## Data Availability

The datasets used and/or analyzed during the current study are available from the corresponding author on reasonable request.

## Abbreviations

MH-GRID: Minority Health Genomics and Translational Research Bio-Repository Database
CES-D: Center for Epidemiologic Studies Depression Scale
PSS: Perceived Stress Scale
GED: Graduate Equivalency Diploma
