# Supplementary material for: Association of active coping to unfair treatment with perceived stress and depressive symptoms in African Americans: mh-grid study
Source: BMC Psychiatry. 2022 Feb 21;22:134. doi: 10.1186/s12888-022-03772-y (PMC8862227; doi:10.1186/s12888-022-03772-y)
Supplement: Supplementary file 1 — Additional file 1. [file 12888_2022_3772_MOESM1_ESM.docx]

**Table S1.** Descriptive characteristics stratified by employment status, MH-GRID ^a^, N = 376

| **Characteristics** | **Employed**  **(N=243)** | **Unemployed**  **(N = 149)** | **P value** |
| --- | --- | --- | --- |
| Age, mean years (std^b^) | 45.38 (6.97) | 46.58 (6.15) | 0.08 |
| Sex |  |  |  |
| Women | 70.46% | 29.54% | <.0001 |
| Men | 46.76% | 53.24% | 0.45 |
| Education |  |  |  |
| ≤12 years or GED^c^ | 44.20% | 55.80% | 0.17 |
| Some college or technical college | 56.14% | 43.86% | 0.19 |
| College graduate or higher | 86.29% | 13.71% | <.0001 |
| Marital Status |  |  |  |
| Unmarried | 54.51% | 45.49% | 0.17 |
| Married | 73.43% | 26.57% | <.0001 |
| Hypertension, mmHg |  |  |  |
| No | 53.19% | 46.81% | 0.45 |
| Yes | 66.81% | 33.19% | <.0001 |
| Depression Scale Score, mean (std^b^) | 9.80 (9.87) | 16.76 (10.44) | <.0001 |
| Perceived Stress Scale score,  mean (std^b^) | 20.36 (8.89) | 24.07 (7.91) | <.0001 |
| How do you respond if treated unfairly? |  |  |  |
| Talk to Someone About It | 63.78% | 36.22% | <.0001 |
| Keep It To Themselves | 50.94% | 49.06% | 0.89 |

^a^ MH-GRID = Minority Health Genomics and Translational Research Bio-Repository Database

^b^ STD = standard deviation

^c^ GED = Graduate Equivalency Diploma

**Table S2.**  Linear regression of the association of talking following exposure to unfair treatment on self-reported level of depression in African Americans stratified by employment status,
MH-GRID^a^ , N = 376

| **Talking following unfair treatment ^b^** | | | | | |
| --- | --- | --- | --- | --- | --- |
| **Depression**  **Scale** | **Unadjusted** | |  | **Adjusted ^c^** | |
|  | **Employed** | **Unemployed** |  | **Employed** | **Unemployed** |
|  | **B estimate P**  **(SE) ^d^ Value** | **B estimate P**  **(SE)** **^d^  Value** |  | **B estimate P**  **(SE)** **^d^  Value** | **B estimate P**  **(SE)** **^d^  Value** |
| **20-item CES-D Scale ^e^** | -7.93 p $<$.0001  (1.99) | -1.26 p = 0.57  (2.23) |  | -5.74 p $=$.0036  (1.95) | -1.14 p = 0.62  (2.27) |

^a^ MH-GRID = Minority Health Genomics and Translational Research Bio-Repository Database

^b^ Compared to keeping it to themselves

^c^ Adjusted model include age, sex, educational level, marital, employment and hypertension status

^d^ SE = Standard Error

^e^ CES-D Scale = Center for Epidemiologic Studies Depression Scale

**Table S3.**  Linear regression of the association of talking following exposure to unfair treatment on self-reported perceived stress in African Americans stratified by employment status,
MH-GRID^a^ , N = 376

| **Talking following unfair treatment^b^** | | | | | |
| --- | --- | --- | --- | --- | --- |
| **Depression**  **Scale** | **Unadjusted** | |  | **Adjusted^c^** | |
|  | **Employed** | **Unemployed** |  | **Employed** | **Unemployed** |
|  | **B estimate P**  **(SE) ^d^ Value** | **B estimate P**  **(SE)** **^d^  Value** |  | **B estimate P**  **(SE)** **^d^  Value** | **B estimate P**  **(SE)** **^d^ Value** |
| **20-item CES-D Scale ^e^** | -7.31 p $<$.0001  (1.79) | -1.15 p = 0.57  (1.69) |  | -5.63 p $=$.0019  (1.79) | -1.25 p = 0.47  (1.73) |

^a^ MH-GRID = Minority Health Genomics and Translational Research Bio-Repository Database

^b^ Compared to keeping it to themselves

^c^ Adjusted model include age, sex, educational level, marital, employment and hypertension status

^d^ SE = Standard Error

^e^ CES-D Scale = Center for Epidemiologic Studies Depression Scale

There was only a borderline significant interaction with a p value ≤ 0.10 between employment status and talking to other people in response to unfair treatment with the outcome being depressive symptoms (p = 0.0974). Therefore, descriptive results stratified by employment status are presented in table S1 of the supplement. The mean age was 45.38 years old among those who were employed and 46.58 among those who were unemployed. There was a larger proportion of women among those who were employed compared to those who are unemployed (70.46% versus 29.54%, pc< .0001). However, there were more men among those who were unemployed than employed (53.24% versus 46.76%, p = 0.45).

A significantly higher proportion of participants with <12 years or GED were unemployed versus employed (55.80% and 44.20%, p = 0.17, respectively). However, a larger proportion of participants with some college/technical college and a college graduate or higher were employed versus the unemployed (56.14% versus 43.86% and 86.29% versus 13.71%, respectively).

A higher proportion of unmarried and married respondents were employed compared to unemployed (54.51% versus 45.49% and 73.43% versus 26.57%, respectively). However, a higher proportion of respondents with and without hypertension were employed compared to those who were unemployed (66.81% versus 33.19% and 53.19% versus 46.81%, respectively).

The average depression score was 9.80 for those who were employed and 16.76 for those who were unemployed. There was a higher proportion of respondents who talked with someone after experiencing unfair treatment were employed (63.78% vs. 36.22%), however there was an equal proportion of respondents who kept it to themselves between those who were married and unmarried (50.94% vs. 49.06%).
 The results of the association between talking following exposure to unfair treatment and employment status is presented in Table S2 of the supplement. In the unadjusted model, those who were employed and talked to someone experienced a lower level of depression by 7.93 units (β:-7.93, standard error: 1.99, p $<$.0001) and 5.74 units in the adjusted model (β:-5.74, standard error: 1.95, p =.0036).
 There was only a borderline significant interaction with a p value ≤ 0.10 between employment status and talking to other people in response to unfair treatment (p = 0.0761) with the outcome for perceived stress scale. The results of the association between talking following exposure to unfair treatment and employment status is presented in Table S3 of the supplement. In the unadjusted model, those who were employed and talked to someone experienced a lower level of depression by 7.31 units (β:-7.31, standard error: 1.79, p $<$.0001) and 5.63 units in the adjusted model (β:-5.63, standard error: 1.79, p =.0019).
